# Nutritional and Pharmacological Effects on Oxidative Stress in Soft Tissue and Bone Remodeling

**DOI:** 10.1155/2018/4183407

**Published:** 2018-12-27

**Authors:** Benjamin M. Savasky, David P. Mascotti, Naren Patel, Edgardo Rodriguez-Collazo

**Affiliations:** ^1^University Hospitals, 27100 Chardon Rd., Richmond Heights, OH 4414, USA; ^2^John Carroll University, Department of Chemistry, 1 John Carroll Blvd., University Heights, OH 44118, USA; ^3^Foot and Ankle Specialists of Ohio, 7482 Center St. Suite #100, Mentor, OH 44060, USA; ^4^St. Joseph Hospital, Department of Surgery, 875 N. Dearborn St. #400, Chicago, IL 60610, USA

## Abstract

Oxidative damage is the causal link to a multitude of pathologies, such as diabetes, arthritis, neuropathy, heart disease, and asthma. These conditions affect hundreds of millions of people nationwide, and billions worldwide. Even in otherwise healthy individuals, oxidative stress is a natural byproduct of metabolism that is augmented in “healthy” activities such as athletics. In many disease states, the pharmacological agents used to treat these conditions can induce oxidative damage and vitamin depletion. It is underappreciated by many that many of the most common medications prescribed result in oxidative stress. Therefore, physicians need to carefully scrutinize which medications their patients are on before surgery and treatment and during the recovery stage to obtain optimal healing results. We provide a review of the current literature of how oxidative damage and inflammation are linked to bone damage, Charcot neuroarthropathy, delayed wound healing, diabetic complications, and delayed flap consolidation. Where available, antioxidant intervention literature is offered to offset these conditions.

## 1. Background

### 1.1. General Oxidative Stress

Reactive oxygen species (ROS) consist of radical and nonradical chemical forms. Oxidative stress (OS) is present when ROS cannot be adequately balanced by the level of antioxidants. This imbalance can occur due to many factors including aging and hormonal changes, radiation exposure, certain drug therapies, certain diseases, and physiological events and an increase of metabolic activity/physical exercise [1–3]. ROS are also produced during normal cellular metabolism following the activation of various enzymes such as NADPH oxidase, mitochondrial oxidases, and superoxide dismutase [4, 5]. ROS levels are also pivotal to induce cell signaling which has a role in cell proliferation, differentiation, apoptosis, and inflammation [2, 6].

Three lines of defense are needed to protect cells from the damaging effects of OS: low molecular weight ROS scavengers, antioxidative enzymes, and proteases for proteolytic degradation of irreversibly damaged proteins. Some of these low molecular weight antioxidants are various vitamins and minerals, co-enzyme Q10 (CoQ10), thiol compounds such as glutathione (GSH), alpha-lipoic acid, *N*-acetyl cysteine, and polyphenols. The ratio of the reduced form of glutathione (GSH) to the oxidized form of glutathione (GSSG) is an important measure of oxidative stress [7, 8]. Further, GSH has the ability to positively influence the activity of certain transcription factors [1, 9, 10] while other reduced thiol forms can act as antioxidant [11] scavengers and regenerate other antioxidants [12, 13]. OS can also damage cell membranes and proteins which can decrease organ function and result in systemic events [13], resulting in nonunion bone fractures, delayed union bone fractures, poor skin viability, poor flap viability, and poor arterial viability. Therefore, understanding how the adverse effects of OS could be abolished by altering the nutritional status of patients is paramount.

A side effect of drug therapy, prescription and over the counter, is depletion of vitamins, minerals, and antioxidants [14]. Many of these depletions affect antioxidants directly, but all may influence overall physiology stressing antioxidant defenses. For instance, corticosteroids function to depress the immune response; however, it leads to increased OS [15]. This may be a function of depletion of melatonin, leading to sleep disruption, and depletion of selenium, which directly relates to glutathione peroxidase activity.

Table 1 indicates the extent of micronutrient depletion that often accompanies both prescription and OTC medication ([16, 17] and references in each). The effect of this depletion has many far-reaching effects, of particular interest to this study, delayed wound healing [18]. An often overlooked weapon in recovery from bone, wound, and surgical tissue damage is repletion of these micronutrients. Few products replete multiple depletions. One notable exception would be Red Ox+.

Herein, we provide a review of the role of oxidative damage in bone remodeling and wound healing, and micronutrient (especially antioxidant) adjunctive therapy and how this may positively affect limb lengthening and microsurgical reconstruction.

### 1.2. Bone Remodeling

Current research demonstrates that oxidative stress can negatively impact bone remodeling [19–22]. Bone remodeling is a complex cycle, which lasts approximately 6 months, and harbors three main cells (osteoclasts, osteoblasts, and osteocytes), which under normal circumstances will be harmoniously regulated with the aid of cytokines, growth factors, and hormones. Oxidative stress activates the differentiation of pre-osteoclasts to adult osteoclasts while inducing apoptosis of osteoblasts and osteocytes and augmenting bone resorption [3, 21].

Oxidative stress has also been shown to upregulate RANKL and downregulate osteoprotegerin. Osteoprotegerin is a decoy receptor for the RANK by competing with RANKL. Osteoprotegerin is directly correlated with estrogen levels [23]. When estrogen levels are low, osteoprotegerin is also low which leads to increased bone resorption. RANKL binds to RANK, which triggers differentiation and activation of osteoclasts. This system is balanced by the relative expression of osteoprotegerin to RANKL, which are highly regulated by many factors including hormones, immune signals, and growth factors. An overexpression of RANKL can cause an overproduction and activation of osteoclasts as shown in primary cell culture from human samples as well as immortal cell line models [9, 13, 24].

Recent literature has displayed that osteocytes constitute 90% of the bone cell population and are essential for bone remodeling. Oxidative stress causes osteocyte apoptosis. These apoptotic osteocytes release sclerostin. Sclerostin is a protein that blocks bone formation by osteoblasts by binding to the Wnt co-receptors and low-density lipoprotein receptors (LRP) 4, 5, and 6 [25, 26]. This decreases the Wnt signaling pathway which, when active, stimulates osteoprotegerin [27–30]. Thus, the outcome of sclerostin upregulation of bone resorption is due, at least in part, by inhibiting the Wnt signaling pathway. Nitrogen bisphosphonates are widely used to control osteoporosis. However, they may contribute to increased OS, particularly when coupled with comorbidities such as diabetes mellitus (DM) and cancer [31]. Thus, the beneficial use of nitrogen bisphosphonates on bone density may also lead to potential negative side effects during high dose or long-term use in cases such as cancer treatment. In these cases, outcomes such as osteonecrosis and atypical long bone fractures have been observed [32]. This may be linked to the OS caused by the nitrogen bisphosphonates and/or their depletion of CoQ10. For instance, it has been found that nitrogen bisphosphonates, as well as statins, inhibit cellular synthesis of the antioxidant CoQ10 [33–35]. Nitrogen bisphosphonate use has also been shown to decrease serum antioxidant vitamin E and CoQ10 levels in postmenopausal women [35]. CoQ10 has been found to reverse spinal cord injury osteoporosis in rats by restoring bone mineral density and bone mineral content while increasing SOD [36]. CoQ10 was also shown to upregulate osteoblast-specific gene core-binding factor alpha 1 [36] and reduce osteoclastogenesis mediated through RANKL while also reducing inflammation in rheumatoid arthritic mice and augmenting the number of T-regulatory cells which directly decreases the number of T-helper 17 cells which are inflammatory [37]. T-regulatory cells are immunosuppressive and help maintain tolerance to self-antigens by preventing autoimmune diseases. Antioxidants, particularly CoQ10, would seem to be a protective adjunct therapy for each of the conditions outlined above.

### 1.3. Wound Healing

**Figure 1:**
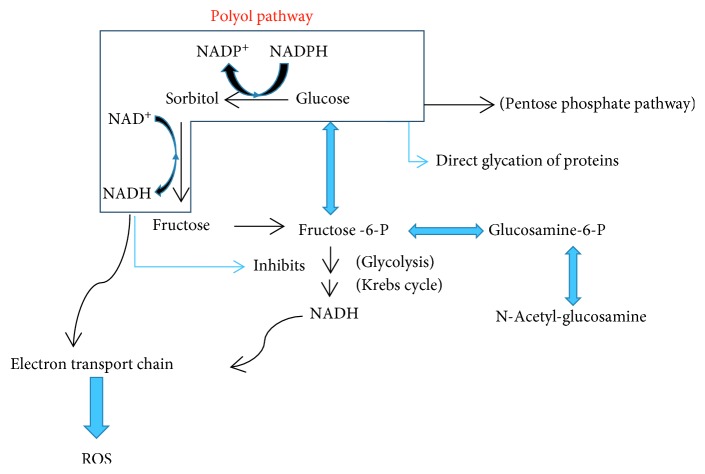
Metabolic fates of glucose relevant to the current focus.

Under normal circumstances, cells utilize glucose for energy via glycolysis which commences using the enzyme hexokinase. However, it has been long known that glucose challenge induces ROS production [38]. There are other fates of glucose such as the pentose phosphate pathway which is generally employed to generate NADPH reducing power and is able to create ribose-5-phosphate for nucleic acid synthesis (Figure 1).

Glucose may also react with aldose reductase in what is known as the polyol pathway to form sorbitol using NADPH as a reductant. Subsequently, the sorbitol is oxidized to fructose by catalysis with sorbitol dehydrogenase and production of NADH. This, in turn, slows glycolysis by inhibiting glyceraldehyde-3-dehydrogenase which prolongs the diversion of glucose away from glycolysis toward the polyol pathway [39]. Furthermore, the high NADH levels induce NADH oxidase activity which may form superoxide radicals as a byproduct [40]. When blood glucose is normal (about 100 mg/dl), aldose reductase has a low affinity for glucose which limits the polyol pathway utilization. However, in diabetics, particularly in chronically poorly controlled glucose patients, glucose levels saturate hexokinase (the first step in glycolysis) and the polyol pathway is engaged. In these chronic hyperglycemic conditions where the polyol pathway is active (Figure 1), there is a decrease in reduced NADPH which leads to decreased synthesis of reduced glutathione, nitric oxide, myo-inositol, and taurine, all of which are important for proper nerve function. Exacerbating this consumption of NADPH in diabetics is lower glucose-6-phosphate dehydrogenase (rate-limiting step in pentose phosphate pathway) activity which prevents NADPH from being produced as demonstrated in a rat model [41].

Reduced glutathione maintains glutathione peroxidase, vitamin C and E in their reduced forms, as well as facilitates proper DNA and protein synthesis [42] and has a vital function in iron metabolism. The decreased NADPH levels due to the polyol pathway compromise these vital reductive functions. Glutathione peroxidase is essential to limiting ROS. The increased sorbitol will augment the production of advanced glycation endproducts (AGEs), reducing kidney function and inducing an inflammatory response while increasing the hemoglobin A1C (HbA1c). This increase in ROS leads to leukocyte adhesion and immigration of inflammatory mediators into the wound bed [43], inhibits re-epithelialization [44], and favors a rich biofilm which may harbor anaerobic bacteria not found in culture [45, 46]. Matrix metalloproteinases (MMPs) are capable of degrading extracellular matrix proteins, releasing apoptotic ligands and are increased in chronic wounds. They are regulated by tissue inhibitors of metalloproteinases. Gencer et al. showed that the expression of MMPs was increased in cells exposed to H_2_O_2_, and that removal of the oxidative stress decreased the expression of the MMPs [47]. Oxidized low-density lipoproteins were also shown to activate MMPs [48]. This appears to offer an intuitive intervention wherein topical antioxidants in conjunction with dietary or otherwise internal antioxidants could limit the amount of tissue damage from damage including surgical.

In a study by Dhall et al., they used the db/db diabetic mouse model of impaired healing and demonstrated that the diabetic mice were subject to chronic wounds with an abundance of free radicals. They then showed that they could reverse this chronicity with the antioxidants vitamin E and *N*-acetylcysteine by decreasing wound healing time, decreasing biofilm concentration, and increasing sensitivity to antibiotics while granulation tissue was formed with proper collagen deposition and remodeling [49]. In fact, diabetic mice, with OS, took over 100 days for their wounds to heal while wild-type mice without OS took on average 15 days for their wounds to heal [49]. When antioxidants were administered, the diabetic mice with OS took only 53 days on average for their wounds to heal [49]. Christman et al. showed that, for each 1.0% point increase in HbA1c, above 7.0%, the wound-area healing rate decreased by 0.028 cm^2^/day in humans with diabetes [50], and with the addition of antioxidants, the HbA1c was able to decrease [51]. The Akt pathway is a signal transduction pathway that promotes cell survival including cell proliferation, cell migration, and angiogenesis. In diabetics with elevated glucose and OS, Akt phosphorylation and gingival wound healing was shown to be impaired. When supplemented with *N*-acetyl-L-cysteine, Akt phosphorylation and gingival wound healing improved as did fibroblast proliferation and migration [52]. Toshiki et al. demonstrated in a rat model that they could significantly increase soft tissue wound healing in the mouth with application of CoQ10. They found that CoQ10 increased the expression of TIMP-1 and FGF while decreasing the expression of MMP-3 [53].

Prolonged OS can also produce psychological maladies including noncompliance and general compromised emotional status [54]. Oxidative damage may also be at the root of some of other psychological maladies such as autism [55]. Patient noncompliance such as not showing up for appointments, inconsistency in taking the medication, or not understanding the severity of their conditions may be due to these psychological OS-driven deficits.

### 1.4. Charcot Neuroarthropathy

Charcot neuroarthropathy (CNA) is caused by an interaction between diabetes, neuropathy, and an inflammatory response [56] which results in bone lysis, mircobone damage, and bone deformity. The “French Theory” (neurovascular theory) suggests that CNA results from a damaged central nervous system which leads to uncontrolled blood flow and prolonged inflammation while the “Germanic Theory” suggests CNA results from trauma secondary to neuropathy which leads to inflammation.

In either case, OS causes the progression of inflammation eventually leading to bone lysis, microfracture, and bone deformity. AGEs are known to be augmented in CNA patients [57]. AGEs are produced during OS [58] and cause apoptosis of osteoblasts [59]. Receptor for advanced glycation endproducts (RAGE) is a transmembrane receptor that binds to AGEs and has been linked to number of neurodegenerative diseases such amyotrophic lateral sclerosis; Alzheimer's, Parkinson's, Huntington's, and Creutzfeldt-Jakob diseases; depression; and CNA [60, 61]. RAGE is known to increase the activity of RANKL leading to osteoclastogenesis in bone [62] and has been associated with atherosclerotic lesions and vascular calcification by increasing the expression of bone morphogenetic protein 4 in arteries [63, 64] and is thought to cause a phenotypic switch of VSMCs to an osteoblast-like phenotype [65]. This pathway is upregulated in CNA [66].

Vascular calcification (VC) is linked to CNA and is associated with OS [67]. VC is four times more likely to be present in diabetic patients then healthy subjects [68], and VC is a significant predictor of stroke, amputation, and myocardial infarction [69]. OS potentiates inflammation by augmenting tumor necrosis factor alpha (TNF-*α*), interleukin-6 (IL-6), and C-reactive protein [70]. CNA patients usually present with augmented serum inflammatory markers with a red hot swollen limb [71]. TNF-*α*, IL-6, and C-reactive protein have all been linked to VC [70] and nerve damage [72], indicting that VC and neuropathy are associated with inflammation. Diabetes is associated with an increase rate of lipoperoxidation [73], while VC is associated with an increased number of oxidized LDLs [74]. Excess mitochondrial ROS are also known to cause VC [29] and are the main cause of nerve damage in diabetics with good glucose control [75].

Brodeur et al. demonstrated that they could decrease calcification in the femoral artery of diabetic rats with the antioxidants: 4-hydroxy-tempol, alpha-lipoic acid, and apocynin. However, only apocynin significantly reduced femoral artery calcification [76]. Kim et al. found that alpha-lipoic acid significantly decreased aortic calcification in mice by inhibiting VSMC apoptosis by preserving mitochondrial function [77].

Related to the DM-induced CNA describe above, vitamin D has been shown by a number of methods to protect pancreatic beta cells from oxidative stress by inducing endogenous antioxidant pathways [78]. Therefore, drug-induced depletion of vitamin D would likely compromise pancreatic stress response.

### 1.5. Flap Consolidation

Numerous studies have shown that oxidative stress can cause inflammatory changes causing decreased blood supply resulting in flap necrosis [79–81]. The increase in OS can cause ischemia reperfusion injury. Necrosis to the distal region of random flaps is a major problem. Ischemia reperfusion injury occurs when the reperfusion creates and harbors oxygen-derived free radicals which are deleterious to the tissues [82]. This is due to increased Fenton chemistry that accompanies reoxidation of iron [83]. Flaps in diabetics present their own risk as OS, an important factor in diabetes mellitus (DM) complications, is already significantly elevated in this population [58] and diminishes the microvascular supply resulting in necrosis [84]. DM also increases AGEs which damage tissue structure which decreases perfusion [85, 86]. One important clinical feature of decreased perfusion, common in diabetics, would be erectile dysfunction (ED) [87, 88]. Antioxidant therapy shows great promise in reversing or preventing ED concurrent with improved blood flow [89, 90].

Calcitriol, a metabolite of vitamin D, is an antioxidant known that has many anti-inflammatory properties. In a random skin flap model, using rats, calcitriol was shown to increase SOD, thereby decreasing OS. This decrease in OS was shown to reduce inflammation and upregulate vascular endothelial growth factor in the flaps which significantly increased flap survival by decreasing edema and increasing angiogenesis [91]. Ufuk et al. showed that random flaps in DM mice exhibited greater OS and necrosis compared to controls while supplementing with long-term antioxidant vitamin E reduced OS and necrosis while increasing hyalinization of arterioles in the flaps [92]. In another study, aminoguanidine, an antioxidant which is known to halt AGEs, was given to DM rats before skin flaps were elevated. Aminoguanidine administration significantly increased flap viability in the diabetic rats by decreasing necrosis [93]. The antioxidants from grape seed extract (proanthocyanidin) and tomato extract (lycopene) were also shown to significantly decrease flap necrosis and inflammation when given two weeks before surgery and two weeks prior surgery [94] in rats. Naringin, the antioxidant found in citrus fruits, was found to significantly increase flap survival by increasing VEGF and SOD while decreasing TNF-*α* and IL-6 [95]. CoQ10 was also shown to significantly increase flap viability in rats while the highest serum level of CoQ10 was obtained with oral administration [96].

## 2. Discussion

Although antioxidants may not cure the root causes of the conditions explored above, many studies have shown that mitigation of the inflammatory products may allow for host recovery and more limited damage [13, 18, 36, 37, 42, 49, 51, 78, 81, 84, 86, 89, 90, 92–94, 96]. Furthermore, starting patients on antioxidants prophylactically could prevent damage from starting. One example would be the vitamin D protection of pancreatic beta cells referred to above [78]. Others would be CoQ10 upregulating osteoblast activity in bone remodeling [36, 37] or antioxidants improving mental disturbances such as autistic behavior, also referred to above [55].

The seven well-known signs of oxidative stress are increased fatigue, memory loss and/or brain fog, muscle and/or joint pain, wrinkles and grey hair, decreased eye sight, headaches and sensitivity to noise, and susceptibility to infections. However, we noted that there are many more signs and symptoms which start at the cellular level and are less obvious for a physician to diagnose such as increased inflammatory mediators resulting in Charcot neuroarthropathy, delayed wound healing, failed surgical outcomes, erectile dysfunction, and poor bone stock.

Physicians should start to examine the GSH/GSSG levels as this will give you a definitive way to quantify oxidative stress. Choosing organic foods and avoiding nicotine and toxic chemicals may prevent some exogenous radicals from entering our body. However, as we age, it is inevitable that the constant bombardment of these radicals, from even natural internal metabolic pathways, will cause a cumulative damage [1, 4, 83]. Many conditions are also fueled by OS, such as rheumatoid arthritis, osteoporosis, and heart disease. We have also referenced evidence that diabetics are subject to OS when increased levels of glucose will enter the polyol pathway [41, 42]. However, we have yet to scrutinize why some individuals with well-controlled blood glucose levels and HbA1c's in the proper range develop neuropathy, Charcot neuroarthropathy, and chronic wounds while some uncontrolled diabetics never have complications. Perhaps, these patients already consume a diet low in oxidative additives, have fortunate genetics, or have some other unknown mechanism that reduces these radicals.

Even the healthiest athlete is subject to OS as exercise will augment the rate of metabolism as food is processed to provide energy at the cellular level. An inevitable side effect of metabolism is the production of ROS and “free radicals” that can damage DNA and other cellular constituents. This damage can lead to cancer, advanced aging, and as we have displayed and increased susceptibility of delayed wound healing and bone remodeling (stress fractures). Osteoporosis is seen daily, whether it is in the elderly, diabetic, postmenopausal female or a consequence of the female athlete triad; these patients are under extreme OS. Bisphosphonates (Table 1) are usually given to counter osteoporosis but could be causing more harm by depleting calcium-magnesium, phosphorus, and Coq10 [14, 16, 17]. Further, starting these patients on steroids or NSAIDs might decrease the discomfort but will cause OS and could potentially make the condition worse. The classic diabetic patient is on a biguanide and or sulfonylurea which both inhibit CoQ10 (Table 1) and have shown to cause neuropathy. Although the diabetes was the initial cause of the neuropathy, it may well be that the prolonged treatment causes enhanced OS leading to further neuropathy. Furthermore, the diabetic patient with many comorbidities is usually taking a statin, ACE inhibitor, beta-blocker, and perhaps an antibiotic for their chronic wound. All these drugs deplete important nutrients (Table 1), potentiating the OS already present from the disease.

Hemodialysis is another process often associated with chronic diabetes patients that leads to loss of nutrients. Chronic kidney disease, as mentioned above, can be a result of the AGEs reducing kidney function. These hemodialysis patients are already at a disadvantage with the retention of toxins and rapid depletion of antioxidants during hemodialysis. According to Liakopoulos et al., the process of iron infusion, length of hemodialysis therapy, anemia, and the central venous catheter all augment OS [97]. Physicians commonly prescribe CoQ10 therapy for a common statin side effect but ignore other depletions such as vitamins B1, B6, B12, C, calcium, magnesium, phosphorus, potassium, zinc, beta-carotene, and folic acid (Table 1). Clearly, with common situations such as this, single vitamin or electrolyte therapies would be insufficient. One would require a more robust formula of all the potential vitamins, electrolytes, and antioxidants to replete the patient such as Red Ox+. There are many more drugs that deplete important nutrients that induce OS. Clinicians need to examine the drugs their patients are on and supplement accordingly. This is especially important for the preoperative and postoperative patient as we have shown through many studies that ameliorating OS will aid in bone remodeling, wound and soft tissue healing, and arterial and muscle and soft tissue flap consolidation. The combination of vitamins, minerals, and antioxidants is an essential weapon clinicians should have in their arsenal that needs to be used on a regular basis.

## 3. Conclusion

Oxidative stress is at the heart of a myriad of maladies. Antioxidants are depleted by many of the medications that were intended to solve these health issues. Robust antioxidants at therapeutic levels would be an excellent way to reverse these ill effects, whether caused by medication-related depletion or directly from the disease state. Doctors should note patient medication profiles and add appropriate supplements accordingly.

More clinical outcomes of antioxidant and vitamin therapy to offset the depletion caused by pharmaceutical intervention should be examined, particularly with respect to markers such as oxidative stress levels, bone density, surgical and wound healing rates, and complete blood cell counts, as well as neuropathy.

## Figures and Tables

**Table 1:** Effects of prescription and over-the-counter medications on micronutrient levels in patients, including vitamins, minerals, and antioxidants ([16, 17] and references in each).

Drug class	Nutrients depleted
*Prescription medications*	
Antibiotics (fluoroquinolones): Levaquin, Avelox, Cipro, Floxin, Noroxin, Penetrex, Trovan	Biotin, B1, B2, B3, B6, B12, zinc, and healthy intestinal bacteria
Antibiotics (macrolides): erythromycin, azithromycin, Biaxin, Zithromax	Healthy intestinal bacteria, B1, B2, B3, B6, and B12
Antibiotics (penicillins): amoxicillin, Amoxil, Trimox, penicillin	Healthy intestinal bacteria, B1, B2, B3, B6, B12, vitamin k, folic acid, biotin, and inositol
ACE inhibitors: lisinopril, Altace, Accupril, Capoten, Prinivil, Zestril, Vasotec	Zinc
Beta-2 adrenergic receptor agonists: albuterol aerosol, Brethine, Proventil, Tornalate, Ventolin, Xopenex	Potassium and possibly calcium-magnesium and phosphorus
Beta-blockers: atenolol, Corgard, Lopressor, Tenormin, Toprol XL, metoprolol	Coenzyme Q10, chromium, and melatonin
Biguanides: metformin, Glucophage	Folic acid, B12
Bisphosphonates: Fosamax, Actonel, Boniva, Didronel, Skelid	Calcium-magnesium and phosphorus
Calcium channel blockers: amlodipine, felodipine, nifedipine, nimodipine, nisoldipine	Vitamin D
Cardiac glycosides: digoxin, Digitek, Lanoxin, Lanoxicaps	Calcium-magnesium, phosphorus, potassium, and B1
Conjugated estrogens: Premarin hormone replacement therapy, birth control pills	B6, vitamin D, calcium-magnesium, zinc, folic acid, and B12
Corticosteroids: Flonase, Beclovent, Beconase, QVar, Vancenase, Vanceril, prednisone, Deltasone, Celestone, Cortisone, Cortef, Cortone, dexamethasone, Decadron, Hydrocortone, Medrol, methylprednisolone	Beta-carotene, B6, folic acid, vitamin C, vitamin D, calcium-magnesium, potassium, selenium, zinc, and melatonin
Loop diuretics: furosemide, Lasix, ethacrynic acid, Edecrin, Bumex	B1, B6, vitamin C, calcium-magnesium, phosphorus, potassium, and zinc
Opiates: hydrocodone/acetaminophen, oxycodone	Folic acid, vitamin C, iron, and potassium
Potassium sparing diuretics: amiloride, spironolactone, triamterene, aldactone, dyazide, Dyrenium, Maxzide	Calcium, magnesium, and phosphorus
Proton pump inhibitors: omeprazole, Prilosec, Prevacid, Nexium, Protonix, Aciphex	Beta-carotene, B1, B12, folic acid, calcium, and zinc
Statins: Lipitor, Crestor, Lescol, Pravachol, Zocor, Mevacor	Coenzyme Q10
Sulfonylurea: glyburide, glipizide, glimepiride, Amaryl, Diabeta, Glucotrol, Glynase, Micronase	Coenzyme Q10
Thiazide diuretics: hydrochlorothiazide	Vitamin D, calcium-magnesium, phosphorus, potassium, zinc, and coenzyme Q10
Tricyclic antidepressants: Amitriptyline, clomipramine, doxepin, imipramine, Anafranil, Asendin, Elavil, Tofranil, Vivactil	Coenzyme Q10, B2, and sodium

*Over-the-counter medications*	
Acetaminophen: Tylenol	Coenzyme Q10 and glutathione
Antacids: Amphojel, Basaljel, aluminum hydroxide plus magnesium, Gaviscon, Gelusil, Maalox, Mylanta	Beta-carotene, folic acid, vitamin D, calcium-magnesium, chromium, iron, zinc, and phosphorus
Aspirin	Folic acid, vitamin C, iron, potassium, and zinc
Laxatives with bisacodyl: Carter's little pills, Correctol, Dulcolax, Feen-A-Mint, PMS-Bisacodyl	Calcium and potassium
NSAIDs: ibuprofen, naproxen	Folic acid
OTC proton-pump inhibitors: famotidine, pepcid, Tagamet, Zantac	Folic acid, B1, B12, vitamin D, calcium, iron, and zinc
